# Cell-Type Specific Channelopathies in the Prefrontal Cortex of the *fmr1-/y* Mouse Model of Fragile X Syndrome^1,2,3^

**DOI:** 10.1523/ENEURO.0114-15.2015

**Published:** 2015-11-17

**Authors:** Brian E. Kalmbach, Daniel Johnston, Darrin H. Brager

**Affiliations:** Center for Learning and Memory, The University of Texas at Austin, C7000, Austin, Texas 78712

**Keywords:** dendrite, excitability, fragile X syndrome, ion channel, prefrontal cortex

## Abstract

Fragile X syndrome (FXS) is caused by transcriptional silencing of the *fmr1* gene resulting in the loss of fragile X mental retardation protein (FMRP) expression. FXS patients display several behavioral phenotypes associated with prefrontal cortex (PFC) dysfunction. Voltage-gated ion channels, some of which are regulated by FMRP, heavily influence PFC neuron function. Although there is evidence for brain region-specific alterations to the function a single type of ion channel in FXS, it is unclear whether subtypes of principal neurons within a brain region are affected uniformly. We tested for alterations to ion channels critical in regulating neural excitability in two subtypes of prefrontal L5 pyramidal neurons. Using somatic and dendritic patch-clamp recordings, we provide evidence that the functional expression of h-channels (*I*_h_) is down-regulated, whereas A-type K^+^ channel function is up-regulated in pyramidal tract-projecting (PT) neurons in the *fmr1-/y* mouse PFC. This is the opposite pattern of results from published findings from hippocampus where *I*_h_ is up-regulated and A-type K^+^ channel function is down-regulated. Additionally, we find that somatic Kv1-mediated current is down-regulated, resulting in increased excitability of *fmr1-/y* PT neurons. Importantly, these h- and K^+^ channel differences do not extend to neighboring intratelencephalic-projecting neurons. Thus, the absence of FMRP has divergent effects on the function of individual types of ion channels not only between brain regions, but also variable effects across cell types within the same brain region. Given the importance of ion channels in regulating neural circuits, these results suggest cell-type-specific phenotypes for the disease.

## Significance Statement

Voltage-gated ion channels regulate the excitability of neurons and are altered in fragile X syndrome (FXS), the most common form of inherited mental retardation. In the *fmr1-/y* mouse model of FXS, we found neuron-type-specific alterations in the function of a group of ion channels within the prefrontal cortex, a brain region associated with many cognitive deficits in FXS. Because of these alterations, neurons with subcortical projections display enhanced excitability while those without subcortical projections do not. This finding highlights the need to understand FXS and tailor its treatment in a cell-type-specific manner.

## Introduction

Fragile X syndrome (FXS) is the most common form of inherited mental retardation and the leading identified monogenic cause of autism. Fragile X mental retardation protein (FMRP), the protein absent in FXS, regulates neuron function via multiple mechanisms, including protein–protein interactions, translational control, and protein trafficking (Hagerman et al., 2005; Bhakar et al., 2012; Santoro et al., 2012; Brager and Johnston, 2014). FMRP is capable of binding to mRNA for several ion channels and is known to interact with ion channel-associated proteins (Brown et al., 2010; Darnell et al., 2011; Brager and Johnston, 2014). Although there is evidence for brain region-specific alterations to channel function in FXS, (for review, see Contractor et al., 2015), it is unclear whether these effects are uniform across neuron types within a brain region. Here, we provide evidence for neuron-type-specific alterations to ion channel function within the prefrontal cortex (PFC) of fmr1-/y mouse.

Neuronal dysfunction in the *fmr1-/y* mouse can be attributed to differences in several types of ion channels (Meredith et al., 2007; Strumbos et al., 2010; Brager et al., 2012; Deng et al., 2013; Routh et al., 2013; Zhang et al., 2014). In some cases, reports are conflicting as to the effect of the loss of FMRP on a given type of ion channel. Some have reported an increase (Lee et al., 2011) in Kv4/A-type K^+^ channel expression, whereas others have found a decrease (Gross et al., 2011; Routh et al., 2013). Similarly, h-channels have been reported to be up-regulated in hippocampus (Brager et al., 2012), but down-regulated in L5B neurons of somatosensory cortex (Zhang et al., 2014).

We tested the hypothesis that the absence of FMRP has different effects on the function of a single type of ion channel in PFC, a brain region containing a heterogeneous neuron population and that is implicated in behavioral deficits associated with FXS (Menon et al., 2004; Krueger et al., 2011; Wang et al., 2012; Dembrow and Johnston, 2014). Using current-clamp and outside-out patch-clamp recordings from *fmr1-/y* mice we provide evidence that *I*_h_ (the current mediated by h-channels) is down-regulated in pyramidal tract (PT)-projecting neurons. This is in contrast to hippocampal CA1 pyramidal neurons, but similar to L5 neurons in somatosensory cortex. Using outside-out patch-clamp recordings, we measured three distinct types of potassium currents: rapidly inactivating A-type current, mediated by putative Kv4 channels; a slowly inactivating current, mediated by putative Kv1 channels; and a sustained current. Again, in contrast to hippocampus, we find that the maximum putative Kv4-mediated current is increased in PT neurons. Additionally, the maximum putative Kv1-mediated current is decreased at the soma of PT neurons, resulting in increased excitability. Notably, these somatic K^+^ channel differences do not extend to neighboring intratelencephalic-projecting (IT) neurons within L5. Thus, the absence of FMRP has divergent effects on a given type of ion channel in different cell types, even within a single brain region. These findings highlight the need to understand FXS and its treatment in a neuron type/brain region-specific manner.

## Materials and Methods

### Bead infusions

All procedures involving animals were approved by the Institutional Animal Care and Use Committee. Male wild-type (WT; C57BL/6) and *fmr1-/y* mice were anesthetized with isoflurane (1 − 4% mixed in oxygen) and prepared for stereotaxic injection of retrograde transported fluorescent-labeled microspheres (Lumafluor). Beads (100 nl) were injected into the pons (in millimeters relative to bregma: posterior 4.2; lateral 0.4; ventral 4.5), mPFC (anterior 1.0–1.5; lateral 0.45; ventral 1–2), or striatum (posterior 0.4–1.0; lateral 2.25; ventral 3.5), using a glass pipette (∼15 μm diameter tip) connected to a nanoject II auto-nanoliter injector (Drummond Scientific) at a rate of 23 nl/s. For all injections, the pipette was left in place for 5 min before removing it from the brain. Mice were given analgesics (Carprofen; 5 mg/kg) and recovered for at least 2 d before their use in experiments. Mice that received different colored beads to label IT and PT neurons were anesthetized with a ketamine (100 mg/kg)/xylazine (10 mg/kg) mixture and were perfused through the heart with ice-cold saline consisting of the following (in mm): 2.5 KCl, 1.25 NaH_2_PO_4_, 25 NaHCO_3_, 0.5 CaCl_2_, 7 MgCl_2_, 7 dextrose, 205 sucrose, 1.3 ascorbate, and 3 sodium pyruvate (bubbled with 95% O_2_/5% CO_2_ to maintain pH ∼7.4) followed by 4% paraformaldehyde in 0.1 m phosphate buffer. Three hundred-micrometer-thick coronal slices were visualized with a Zeiss Axio Imager Z2 microscope running Axio Vision software (Carl Zeiss). We looked for clear instances of overlap of green and red puncta across at least four 300 μm sections per animal.

### Slice preparation

Male WT and *fmr1-/y* mice, 8–16 weeks old, were anesthetized with a ketamine (100 mg/kg)/xylazine (10 mg/kg) mixture and were perfused through the heart with ice-cold saline consisting of the following (in mm): 2.5 KCl, 1.25 NaH_2_PO_4_, 25 NaHCO_3_, 0.5 CaCl_2_, 7 MgCl_2_, 7 dextrose, 205 sucrose, 1.3 ascorbate, and 3 sodium pyruvate (bubbled with 95% O_2_/5% CO_2_ to maintain pH ∼7.4). A vibrating tissue slicer (Vibratome 3000, Vibratome) was used to make 300-μm-thick coronal sections. Slices were held for 30 min at 35°C in a chamber filled with artificial CSF (aCSF) consisting of the following (in mm): 125 NaCl, 2.5 KCl, 1.25 NaH_2_PO_4_, 25 NaHCO_3_, 2 CaCl_2_, 2 MgCl_2_, 10 dextrose, and 3 sodium pyruvate (bubbled with 95% O_2_/5% CO_2_) and then at room temperature until the time of recording.

### Electrophysiology

Recordings were made from L5 pyramidal neurons in the dorsal, medial prefrontal cortex ∼1–2 mm anterior to bregma. Slices were placed in a submerged, heated (32–34°C) recording chamber that was continually perfused (1 − 2 ml/min) with bubbled aCSF containing the following (in mm): 125 NaCl, 3.0 KCl, 1.25 NaH_2_PO_4_, 25 NaHCO_3_, 2 CaCl_2_, 1 MgCl_2_, 10 dextrose, 3 sodium pyruvate, 0.025 D-APV, 0.02 DNQX, 0.005 CGP, and 0.002 gabazine. For outside-out recordings, 0.001 mm TTX was added to the aCSF. Slices were viewed with either: (1) a Zeiss Axioskop microscope and differential interference optics, (2) a Zeiss AxioExaminer D microscope and Dodt contrast optics, or (3) a two-photon laser-scanning microscope (Leica SP5 RS) using Dodt contrast. Fluorescent-labeled neurons were visualized using a mercury lamp and a 540/605 nm or 470/502 nm excitation/emission filter set or two-photon excitation at 840 nm. Patch pipettes (4–8 MΩ) were pulled from borosilicate glass and wrapped with Parafilm to reduce capacitance. The pipette solution for all configurations contained the following (in mm): 120 K-gluconate, 16 KCl, 10 HEPES, 8 NaCl, 7 K_2_ phosphocreatine, 0.3 Na-GTP, 4 Mg-ATP, pH 7.3 with KOH. Neurobiotin (Vector Laboratories; 0.1%) was also included for histologic processing. For all experiments involving dendritic recordings and some experiments involving somatic recordings, AlexaFluor 594 (16 μm; Invitrogen) was also included in the internal recording solution to determine the recording location. All drugs were prepared from concentrated stock solutions in water and were obtained from Abcam Pharmaceutical or Tocris.

### Whole-cell recordings

Data were acquired using a Dagan BVC-700 (Dagan) amplifier and custom data acquisition software written using Igor Pro (Wavemetrics) or AxoGraph X (AxoGraph Scientific) data acquisition software. Data were acquired at 10 − 50 kHz, filtered at 5 − 10 kHz, and digitized by an ITC-18 (InstruTech) interface. Pipette capacitance was compensated and the bridge was balanced during each recording. Series resistance was monitored throughout each experiment and was 10 − 25 MΩ for somatic recordings and 15 − 40 MΩ for dendritic recordings. Voltages are not corrected for the liquid-junction potential (estimated as ∼12 mV).

Data were analyzed using either custom analysis software written in Igor Pro or using AxoGraph X. Subthreshold membrane properties were measured at a common membrane potential (−65 mV). Input resistance (*R*_N_) sag and rebound were calculated from the voltage response to a family of 1000 ms current injections (−150 to +50 pA, 20 pA steps. Input resistance (*R*_N_) was calculated from the linear portion of the current−voltage relationship. Voltage sag was defined as the ratio of maximum to steady-state *R*_N_. Rebound slope was calculated from the slope of the rebound amplitude as a function of steady-state membrane potential. The functional membrane time constant was defined as the slow component of a double-exponential fit of the average voltage decay in response to hyperpolarizing current injections (100 − 300 pA, 2 ms). Resonance was determined from the voltage response to a constant amplitude sinusoidal current injection that linearly increased in frequency from 1 − 15 Hz in 15 s. The impedance amplitude profile (ZAP) was constructed from the ratio of the fast Fourier transform of the voltage response to the fast Fourier transform of the current injection. The peak of the ZAP was defined as the resonant frequency. Single action potentials (APs) were elicited using just-threshold current injections of various durations. AP threshold was defined as the voltage where the first derivative first exceeded 20 mV/ms. Simulated synaptic currents were generated with exponentially rising (dendrite = 0.2 ms; soma = 0.3 ms) and decaying (dendrite = 2 ms; soma = 4 ms) waveforms. The amplitude of this waveform was adjusted such that amplitude of the voltage response to a single simulated event was ∼3 mV. For recordings from non-labeled neurons, the presence (>2.2 Hz) or absence of resonance was used to classify neurons into projection types.

### Outside-out recordings

Membrane currents were recorded using an Axopach 200B amplifier (Molecular Devices), sampled at 10 kHz, analog filtered at 2 kHz and digitized by an ITC-18 interface connected to a computer running Axograph X. For K^+^ channels, activation curves were constructed by using depolarizing voltage commands (−70 to 50 mV in 20 mV steps) to activate *I*_K_ from a holding potential of −90 mV. Activation data were fit to a single Boltzmann function using a least-squares program. For voltage commands more hyperpolarized than −10 mV, we extrapolated the slow time constant of the current back to the time equal to the peak of the total current. In this way, the peak of the slow and fast inactivating currents could be separated. The accuracy of this procedure was confirmed using a small set of experiments where the slowly inactivating component was measured directly. Linear leakage and capacitive currents were digitally subtracted by scaling traces at smaller command voltages in which no voltage-dependent current was activated.

### Data analysis

Repeated-measures (RM) ANOVA, between-subject factors ANOVA, mixed factors ANOVA, and *post hoc t* tests were used to test for statistical differences between experimental conditions. Bonferroni correction was used to correct for multiple comparisons. Pearson’s product moment correlation was used to test for statistically significant correlations between variables. Error bars represent SEM. Statistical analyses were performed Prism (Graphpad). Data are presented in the text as mean ± SEM.

## Results

### The organization of L5 projection neurons is maintained in the *fmr1-/y* mouse PFC

Testing for differences in ion channel function is complicated by heterogeneity in pyramidal neuron types within PFC. Pyramidal neurons are present throughout L2–6 and possess distinct morphology, connectivity, and repertoires of ion channels (Molyneaux et al., 2007; Shepherd, 2013; Dembrow and Johnston, 2014). For example, in L5, neurons projecting solely within the telencephalon (IT) possess distinct morphologic and physiologic properties from those of neighboring neurons that project subcortically through the PT (Christophe et al., 2005; Molnár and Cheung, 2006; Hattox and Nelson, 2007; Dembrow et al., 2010; Morishima et al., 2011; Otsuka and Kawaguchi, 2011; Sheets et al., 2011; Avesar and Gulledge, 2012; Gee et al., 2012; Kalmbach et al., 2013; Lee et al., 2014).

Before testing for alterations to physiology, we first asked whether the two general types of L5 projection neurons are found in the *fmr1-/y* mouse medial PFC (mPFC). In WT and *fmr1-/y* mice, we infused red retrograde tracer (Lumuflour beads) into the pontine nuclei to label PT neurons and a green tracer into either the contralateral striatum or contralateral mPFC to label IT neurons. In both genotypes, IT neurons were found throughout L2–6, whereas PT neurons were restricted to L5/6. Within the deep layers, PT and IT neurons could be observed within close proximity to each other at all depths (<20 μm). Furthermore, we did not observe clearly any double-labeled neurons in either genotype (*n* = 2 for each genotype; Fig. 1*A*). Together, these data suggest that in *fmr1-/y* mice, as in WT mice, IT and PT neurons represent two non-overlapping populations of neurons.

**Figure:**
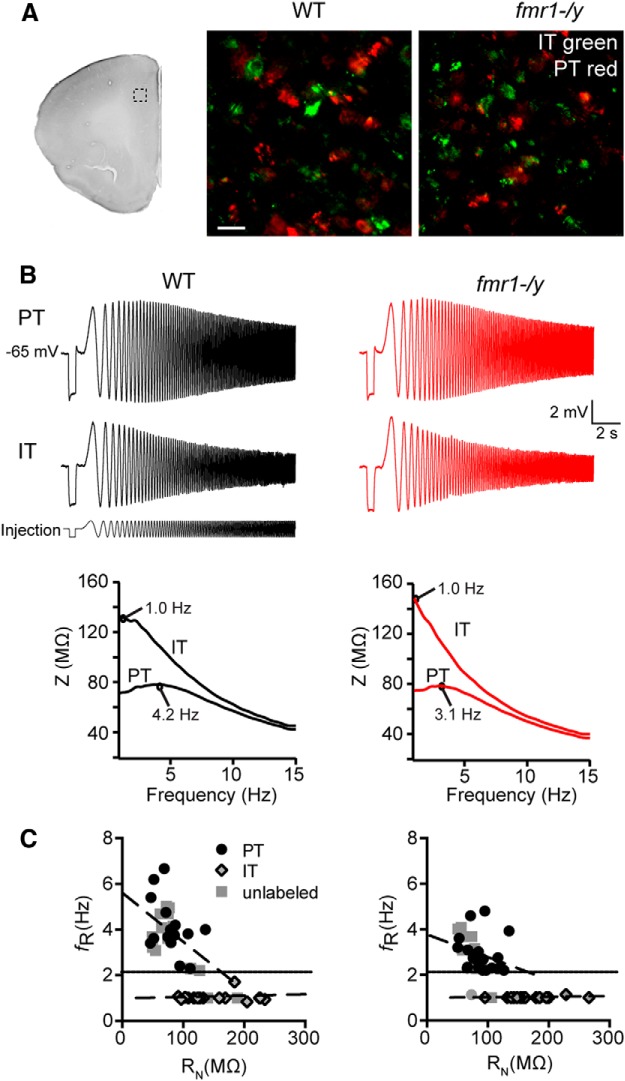
Figure 1**. PT and IT neurons in L5 of mPFC in the *fmr1-/y* mouse. *A***, Coronal section of one hemisphere of mouse mPFC illustrating approximately where neurons where visualized. Retrograde labeled IT and PT neurons in the middle of L5 in WT (left) and *fmr1-/y* mouse mPFC. IT (green) and PT (red) neurons were non-overlapping populations of neurons in both genotypes. Scale bar, 20 μm. ***B***, Response to a chirp current injection in WT (left) and *fmr1-/y* (right) PT and IT neurons and the resultant ZAP for each neuron. ***C***, IT and PT neurons were distinguishable based on intrinsic membrane properties in both genotypes. In both genotypes, PT neurons were resonant, whereas IT neurons were non-resonant. *f*_R_ in PT neurons was negatively correlated with *R*_N_ in both genotypes (*p* < 0.05).

As an additional test, we next asked whether L5 IT and PT neurons in *fmr1-/y* mice could be distinguished based upon their physiologic properties. PT neurons possess conductances that endow them with membrane resonance in the 3–7 Hz range whereas IT neurons are non-resonant. Thus, the presence of resonance can be used to distinguish PT from IT neurons (Dembrow et al., 2010). For these experiments, we made whole-cell current-clamp recordings from the soma of identified L5 PT or IT neurons, as well as nearby unlabeled cells. In both WT (*n* = 15 cells from 6 mice, 4.11 ± 0.32 Hz) and *fmr1-/y* (*n* = 21 cells from 7 mice, 2.79 ± 0.19 Hz) mice, PT neurons displayed membrane resonance whereas IT neurons did not (WT: n = 17 cells from 5 mice, 1.07 ± 0.06 Hz; *fmr1-/y*: *n* = 19 cells from 6 mice, 1.01 ± 0.01 Hz; IT vs PT, *p* < 0.001^a^, ANOVA; Fig. 1*B*,*C*; Table 1). Furthermore, in both genotypes, input resistance (*R*_N_) was significantly higher in PT compared with IT neurons (WT PT = 79.53 ± 6.65 MΩ; WT IT = 112.90 ± 11.78 MΩ; *fmr1-/y* PT = 89.04 ± 5.21 MΩ, *fmr1-/y* IT 171.3 ± 8.73 MΩ; *p* < 0.001^b^, ANOVA; Fig. 1*B*,*C*). We also recorded from non-resonant (WT: *n* = 3 cells from 2 mice; *fmr1-/y*: *n* = 3 from 2 mice) and resonant (WT: *n* = 13 from 4 mice; *fmr1-/y*: *n* = 11 cells from 3 mice) non-labeled neurons with physiologic properties that closely resembled nearby labeled IT and PT neurons, respectively. When these putative IT and PT neurons were grouped with labeled projection neurons for analysis, we found that in both genotypes, PT neuron resonant frequency was inversely correlated with *R*_N_, whereas in IT neurons it was not (Fig. 1*C*; WT PT: *r* ^2^ = 0.22, *p* = 0.01^c^; WT IT: *r* ^2^ = 0.01, *p* = 0.62; *fmr1-/y* PT: *r* ^2^ = 0.12, *p* = 0.05, *fmr1-/y* IT: *r* ^2^ = 0.08, *p* = 0.18). These data are consistent with findings in rat mPFC (Dembrow et al., 2010) and suggest that the organization of projection neurons in L5 of PFC is maintained in the *fmr1-/y* mouse. For our purposes, unlabeled neurons that display resonance will be referred to as PT neurons, whereas those that do not will be referred to as IT neurons.

**Table 1.** Statistical Tests

	**Data**	**Test**	**Measurement/group**	**Power**		**Effect size**
a	Unknown	ANOVA		100		3.28
b	Normal	ANOVA		100		1.21
c	Normal	Pearson correlation	WT PT	65.6		0.28
	Unknown	Pearson correlation	WT IT	6.3		0.01
	Normal	Pearson correlation	*fmr1-/y* PT	40.9		0.14
	Unknown	Pearson correlation	*fmr1-/y* IT	19		0.09
d	Normal	*t* test	*R*N distal	94.5		0.98
	Normal	*t* test	*R*N proximal	84.5		1.14
	Normal	*t* test	*R*N soma	23.2		0.31
e	Unknown	ANOVA	Rebound	98.3		0.37
	Unknown	ANOVA	Sag	99.6		0.39
	Normal	ANOVA	RMP	97.6		0.33
f	Unknown	ANOVA	Resonance	99.9		0.47
	Normal	ANOVA	tau	99.8		0.43
g	Normal	ANOVA	Soma	94		0.79
	Normal	ANOVA	Dendrite	97		0.74
h	Normal	ANOVA	Rn	36		0.22
	Unknown	ANOVA	Sag	12.6		0.11
	Unknown	ANOVA	Rebound	43		0.24
	Unknown	ANOVA	Resonance	6.5		0.05
i	Normal	ANOVA	67.2	0.33		
j	Normal	ANOVA	55.2	0.71		
k	Unknown	*t* test	*R*N	18.1		0.73
	Unknown	*t* test	Sag	2.5		0
	Unknown	*t* test	Rebound	11.3		0.57
	Unknown	*t* test	Resonance	10.4		0.54
	Unknown	*t* test	tau	18.2		0.73
	Unknown	*t* test	RMP	2.9		0.13
	Unknown	*t* test	Summation	6.2		0.38
l	Unknown	ANOVA	5.3	0.08		
m	Unknown	*t* test		98.8		4.80
n	Unknown	*t* test		96.1		1.77
o	Normal	ANOVA	99.9	0.93		
p	Normal	*t* test	94.7	1.50		
q	Normal	*t* test	*I*KA-fast	77.9		1.50
	Normal	*t* test	*I*K-sustained	60.4		1.35
r	Unknown	*t* test	*I*KA-fast	8.1		0.55
	Unknown	*t* test	*I*K-sustained	3.1		0.21
s	Normal	*t* test	*I*KA-fast soma vs dend*fmr1-/y*	79.6		1.74
	Normal	*t* test	*I*KA-fast soma vs dend WT	3.6		0.23
	Normal	*t* test	*I*K-sustained soma v dend*fmr1-/y*	72.5		1.72
	Normal	*t* test	*I*K-sustained soma vs dend WT	8.67		0.58
t	Normal	*t* test	WT soma v dend	92.1		2.20
	Normal	*t* test	KO soma v dend	86.6		1.99
u	Normal	*t* test		51.5		1.14
v	Normal	ANOVA	*I*K-total	5.4		0.03
	Normal	ANOVA	*I*KA-fast	27.4		0.27
	Normal	ANOVA	*I*K-sustained	8.4		0.11
	Normal	ANOVA	*I*K-slow	9.2		0.13
w	Normal	ANOVA		13.8		0.19
x	Unknown	*t* test		85		2.07
y	Unknown	*t* test		99.2		0.20
z	Normal	ANOVA		26.5		0.30
a'	Normal	ANOVA		10		0.15
b'	Unknown	ANOVA	4-AP	100		2.02
Unknown	ANOVA	α DTX	100		5.20	
c'	Unknown	ANOVA		100		3.74
d'	Unknown	*t* test		54		1.76
e'	Unknown	*t* test		3		0.20
f'	Unknown	ANOVA		7.7		0.12
g'	Normal	ANOVA		100		1.50
h'	Normal	*t* test		12	71	0.89
I'	Normal			24	93	1.19
j'	Normal			50	88.9	1.11
k'	Normal			100	81.9	1.00
	Normal			100		0.51
	Normal	ANOVA		71.2		0.18
	Normal	ANOVA		62.6		0.21
k'	Normal	ANOVA	WT	99.9		0.90
l'	Normal		*fmr1-/y*	94.3		0.61
m'	Normal	ANOVA		99.9		0.81
n'	Normal	ANOVA		99.9		0.75
	Normal	ANOVA	WT	10		0.14
o'	Normal	ANOVA	*fmr1-/y*	17.78		0.19
p'	Normal	ANOVA		99.9		1.03
q'	Normal	ANOVA		92.2		0.38

Data structure was tested for normality using Kolmogorov–Smirnoff tests. In cases where the test for normality failed, nonparametric statistic yielded similar results. For some comparisons, the measurement and/or group under comparison is listed for clarity. *Post hoc* power analysis was performed using G × Power (v3.1.9.2, www.Gpower.hhu.de).

### *I*_h_ is reduced in L5 neurons in the *fmr1-/y* mouse mPFC

The intrinsic membrane properties of pyramidal neurons are heavily influenced by the presence of voltage-gated ion channels (Magee, 2000; London and Häusser, 2005; Johnston and Narayanan, 2008). The expression of one class of channel, h-channels, which carry *I*_h_, has been reported to be up-regulated in CA1 but down-regulated in somatosensory cortex in the *fmr1-/y* mouse (Brager et al., 2012; Zhang et al., 2014).

To test for alterations to h-channel function in PFC we measured *I*_h_-sensitive membrane properties (membrane potential – *V*_m_, *R*_N_, time constant – tau, sag, rebound, resonance, and temporal summation; Hutcheon and Yarom, 2000; Robinson and Siegelbaum, 2003; Brager and Johnston, 2007; Narayanan and Johnston, 2007) in WT and *fmr1-/y* neurons. Because h-channel expression in many types of cortical pyramidal neurons increases with distance from the soma (Lörincz et al., 2002; Kole et al., 2006), we performed these measurements at the soma (WT: *n* = 30 cells from 18 mice; *fmr1-/y*: *n* = 35 cells; from 17 mice), proximal dendrite (≤250 μm; WT: *n* = 12 cells from 9 mice; *fmr1-/y*: *n* = 17 cells from 17 mice), and distal dendrite (>250 μm; WT: *n* = 34 cells from 26 mice; *fmr1-/y*: *n* = 23 cells from 17 mice). We focused our initial efforts on PT neurons because they have more pronounced h-channel associated properties (Fig. 1; Dembrow et al., 2010; Sheets et al., 2011; Kalmbach et al., 2013). *R*_N_ was higher at proximal and distal dendritic recording locations (*p* < 0.01^d^) in *fmr1-/y* compared with WT neurons, but this difference was not observed at the soma (*p* = 0.21; Fig. 2*A*,*B*). *Fmr1-/y* neurons were more hyperpolarized, displayed less sag and less rebound than WT neurons, at all recording locations (all *p* < 0.01; Fig. 2*A*,*B* ^e^). Furthermore, the resonant frequency of *fmr1-/y* neurons was lower and the functional membrane time constant longer than WT neurons at all recording locations (*p* < 0.01; Fig. 2*C*,*D* ^f^). Finally, *fmr1-/y* neurons displayed greater temporal summation of simulated synaptic currents at both the soma and distal dendritic recording locations (*p* < 0.001, ANOVA; Fig. 2*E* ^g^). Together, these differences in membrane properties between genotypes suggest that there is a decrease in *I*_h_ at both the soma and dendrite of L5 PT neurons in PFC of *fmr1-/y* mice.

**Figure:**
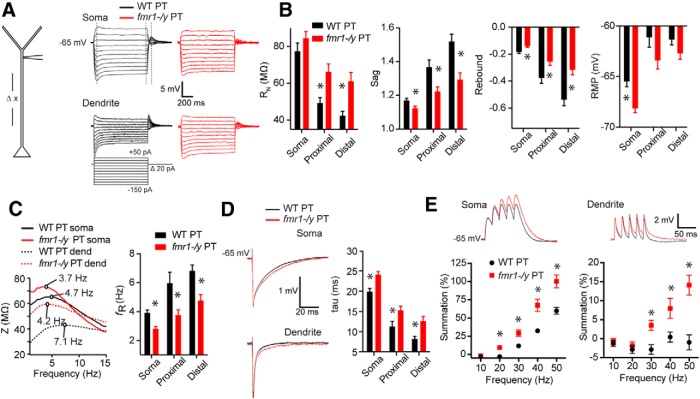
Figure 2**. Altered h-dependent membrane properties in L5 PT neurons of mPFC in the *fmr1-/y* mouse. *A***, Left, Whole-cell recordings were made at various distances from the soma in WT and *fmr1-/y* mouse PT neurons. Right, Example traces from whole-cell recordings from the soma and dendrite of WT and *fmr1-/y* neurons in response to the current injection shown below. All recordings were made at −65 mV ***B***, *R*_N_ was higher in the dendrite, but not soma of *fmr1-/y* neurons compared with WT. In addition, *fmr1-/y* neurons were more hyperpolarized and displayed less sag and rebound at the soma and dendrite compared with WT neurons. ***C***, *fmr1-/y* neurons displayed a lower resonant frequency at the soma and dendrite compared with WT neurons. Sample ZAPs are shown at right. ***D***, The functional membrane time constant was longer in *fmr1-/y* compared with WT neurons, at all recording sites. ***E***, *fmr1-/y* neurons displayed greater temporal summation of simulated synaptic currents compared with WT neurons at the soma and distal dendritic recording locations. *p < 0.01, *post hoc* comparison across genotypes.

To test for differences in IT neurons, we made many of these same measurements at the soma (WT: *n* = 20 cells from 8 mice; *fmr1-/y*: *n* = 22 cells from 10 mice) and dendrite (WT: *n* = 7 cells from 7 mice; 125–270 μm, 192.86 ± 17.52 μm from soma; *fmr1-/y*: *n* = 8 cells from 8 mice; 145–300 μm, 235 ± 20.28 μm from soma). In contrast to PT neurons, the subthreshold properties of IT neurons were largely similar in WT and *fmr1-/y* mice (all comparisons *p* > 0.1; ANOVA; Fig. 3 ^h^). The lone exception was resting membrane potential (RMP) where *fmr1-/y* neurons were more hyperpolarized compared with WT neurons (*p* = 0.02; ANOVA; Fig. 3*A* ^i^). Differences in RMP at the soma persisted in the presence of the h-channel blocker ZD7288 (10 μm; WT: *n* = 6 cells from 4 mice baseline −64.33 ± 1.50 ZD7288 −68.52 ± 0.23; *fmr1-/y*: *n* = 6 cells from 4 mice baseline −69.63 ± 1.56 ZD7288 −72.15 ± 1.48; *p* = 0.04; ANOVA^j^), suggesting that h-channels were not the main source of these differences.

**Figure:**
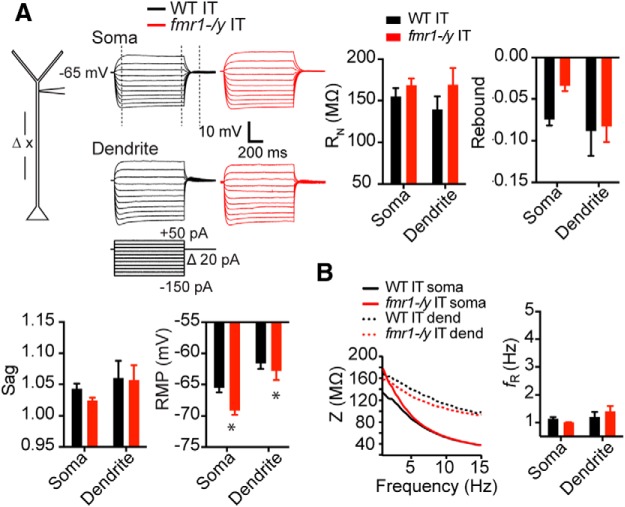
Figure 3**. Subthreshold membrane properties are largely unaltered in IT neurons of PFC in the *fmr1-/y* mouse. *A***, Left, Whole-cell recordings were made at various distances from the soma in WT and *fmr1-/y* mouse IT neurons. Right, Example traces from whole-cell recordings from the soma and dendrite of WT and *fmr1-/y* neurons in response to the current injection shown below. All recordings were made at −65 mV. *R*_N_, sag, and rebound were not different at the soma or dendrite in *fmr1-/y* compared with WT IT neurons. RMP was hyperpolarized in *fmr1-/y* compared with WT IT neurons. ***B***, *fmr1-/y* and WT IT neurons both displayed a similar resonant frequency at the soma and dendrite. *p < 0.05, *post hoc* comparison across genotypes.

To test whether the observed differences in PT neurons were due primarily to h-channels, we compared the effects of the ZD7288 on the properties of PT L5 dendrites in WT versus *fmr1-/y* mice. Application of 10 μm ZD7288 largely eliminated the differences in membrane properties between WT (*n* = 7 cells from 6 mice) and *fmr1-/y* (*n* = 9 cells from 7 mice) neurons measured at a common membrane potential (−65 mV; Fig. 4). There were no statistical differences in *R*_N_ (*p* = 0.12), *V*_m_ (*p* = 0.94), sag (*p* = 0.40), rebound (*p* = 0.08; Fig. 4*A*,*B*), resonant frequency (*p* = 0.37; Fig. 4*C*), tau (*p* = 0.18; Fig. 4*D*), or temporal summation of simulated synaptic input (*p* = 0.30; Fig. 4*E*) in the presence of ZD7288 in WT versus *fmr1-/y* neurons (all *post hoc* comparisons^k^).

**Figure:**
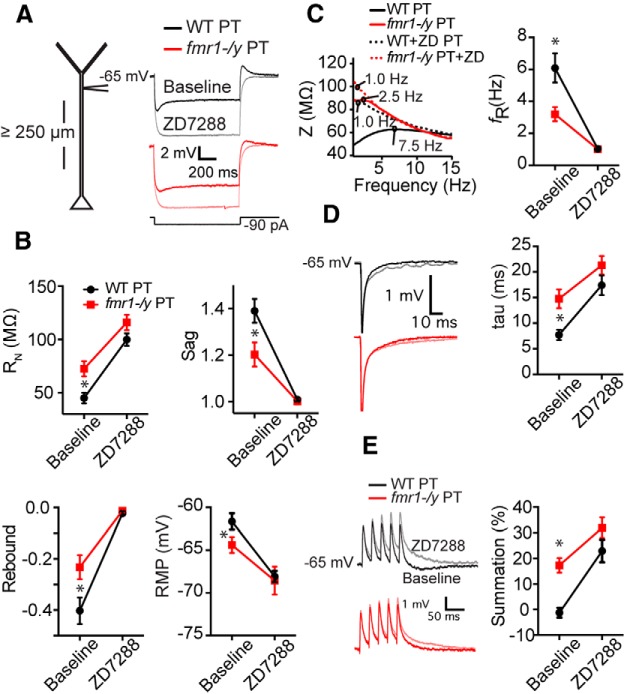
Figure 4**. Differences in membrane properties between WT and *fmr1-/y* neurons are eliminated by the h-channel blocker, ZD7288. *A***, Sample voltage responses to a hyperpolarizing current injection at the distal dendrite of *fmr1-/y* and WT PT neurons. ***B***, ZD7288 (10 μm) eliminated differences in *R*_N_, Sag, rebound and RMP between WT and *fmr1-/y dendrites*. ***C***, ZD7288 also eliminated membrane resonance in both genotypes. ***D***, The functional membrane time constant and temporal summation of simulated synaptic currents delivered at 50 Hz (***E***) was no different between *fmr1-/y* and WT dendrites in the presence of ZD7288. All measurements were performed at −65 mV. *p < 0.01.

Finally, we made outside-out patch-clamp recordings from the distal dendrite of PT neurons to directly compare *I*_h_ in WT (*n* = 4 cells from 3 mice; 225–400 µm, 340 ± 37.6 µm from soma) and *fmr1-/y* (*n* = 5 cells from 5 mice; 250–350, 308 ± 17.7 µm from soma) mice. Voltage steps from a holding potential of −30 to −140 mV elicited an inward current in both genotypes (Fig. 5*A*). The activation of this current was well fit by a double-exponential. The fast and slow time constants were not different between genotypes and both were consistent with *I*_h_ (Fig. 5*B*; *p* = 0.87, mixed-ANOVA^l^; Dougherty et al., 2013). The maximum dendritic *I*_h_ was greater in WT compared with *fmr1-/y* neurons (Fig. 5*C*; *p* = 0.001, *t* test^m^). Finally, the resonant frequency measured during whole-cell before obtaining the outside-out configuration was lower in *fmr1-/y* compared with WT dendrites (Fig. 5*D*; *p* = 0.03, *t* test^n^). These data are consistent with a decrease in the functional expression of h-channels in L5 PT neurons of PFC in the *fmr1-/y* mouse.

**Figure:**
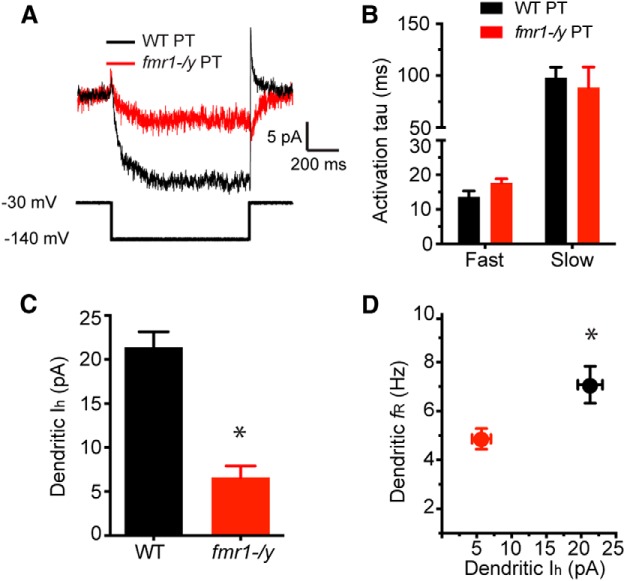
Figure 5**. *I*_h_ is reduced in the dendrites of L5 PT neurons of PFC in the fmr1-/y mouse. *A***, h-Current was measured by stepping from a holding potential of −30 mV to −140 mV for 500 ms. ***B***, There were no genotypic differences in the time constant of activation of *I*_h_. ***C***, Both *I*_h_ and resonance (***D***) were significantly reduced in the dendrite of *fmr1-/y* neurons compared with WT. *p < 0.05

### Alterations to I_K_ in L5 mPFC pyramidal neurons in *fmr1-/y* mice

The observed decrease in *I*_h_ in PT neurons is in contrast to pyramidal neurons in CA1 of the hippocampus (Brager et al., 2012), but is similar to L5 pyramidal neurons in somatosensory cortex (Zhang et al, 2014). Together, these observations suggest that the absence of FMRP can have divergent effects on channel expression/function depending upon brain region or neuron type. A-type K^+^ and h-channel function are affected in opposite directions by several manipulations (Harris-Warrick et al., 1995; Bernard et al., 2004; Frick et al., 2004; Fan et al., 2005; Shin et al., 2008). Thus, we next asked whether the functional expression of A-type K^+^ channels, which is decreased in CA1 pyramidal neurons in the *fmr1-/y* mouse (Routh et al., 2013), is increased in L5 neurons of mPFC and if so, whether the change is restricted to one class of L5 projection neuron.

We used outside-out patch recordings to measure the total K^+^ current at the soma and distal dendrite (≥250 μm) of PT L5 neurons in WT and *fmr1-/y* mice (Fig. 6*A*). The current elicited by step depolarization to 50 mV from a holding potential of −90 mV contained both a transient and a sustained component (*I*_K-total_; Fig. 6*A1*). Macroscopic K^+^ currents in L5 neurons in granular cortex are composed of distinct channels with fast and slow inactivation kinetics (Bekkers, 2000; Korngreen and Sakmann, 2000). To test whether PT neurons in mPFC possess similar diversity in K^+^ currents, we used the voltage-dependent properties of the putative channels underlying each of these components to isolate the individual currents. A brief (100 ms) prepulse to −20mV was used to inactivate the fast-inactivating current without significantly affecting either the sustained or slowly inactivating current (Fig. 6*A2*). Subtracting the resulting current from total *I*_K_ revealed a rapidly inactivating K^+^ current (*I*_KA-fast_; Fig. 6*A4*). Step depolarization to 50 mV from a holding potential of −20 mV, to inactivate all transient K^+^ currents, revealed a sustained current (*I*_K-sustained_; Fig. 6*A3*). Subtracting *I*_K-sustained_ from the current in Fig. 6*A2* revealed a slowly inactivating K^+^ current (*I*_K-slow_, Fig. 6*A5*). Thus, we were able to isolate three kinetically distinct outward currents: *I*_K-sustained_, *I*_KA-fast_, and *I*_K-slow_ (Fig. 6*A*).

**Figure:**
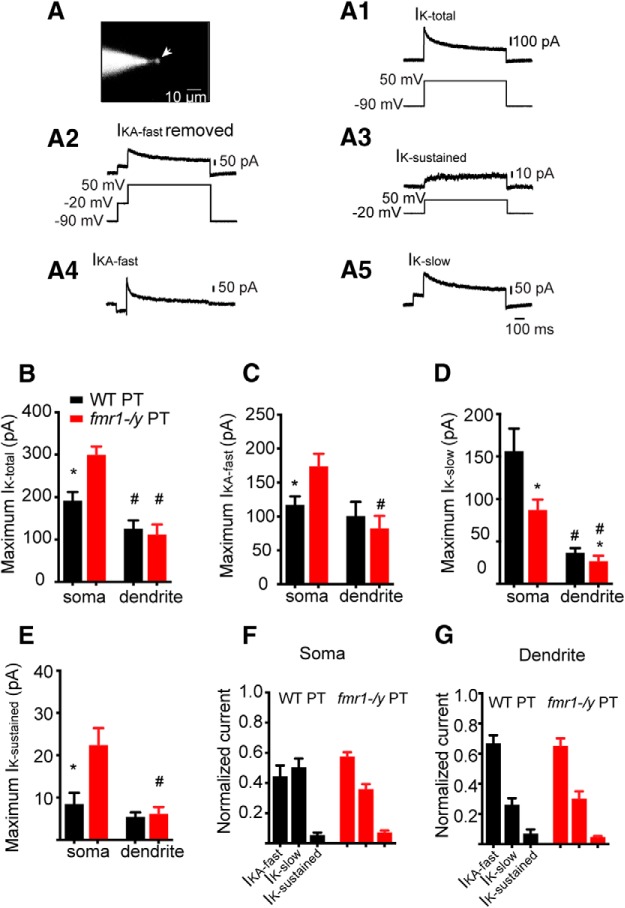
Figure 6**. Differences in *I*_K_ in PT neurons in *fmr1-/y* versus WT mice. *A***, Outside-out patch from a PT neuron visualized through fluorescence microscopy. *I*_K-total_ was measured by stepping to +50 mV from a holding potential of −90mV. A prepulse to −20 mV was used to inactivate fast inactivating *I*_KA_. *I*_K-sustained_ was obtained by stepping from a holding potential of −20 mV to +50 mV. *I*_KA-fast_ was obtained by subtracting the current in ***A2***, from the total current in ***A1***. *I*_K-slow_ was obtained by subtracting the sustained current in ***A3*** from the current obtained in ***A2***. ***B***, Maximum *I*_K-total_ at the soma is greater in *fmr1-/y* PT neurons compared with WT neurons. ***C***, Maximum *I*_KA-fast_ at the soma is greater in *fmr1-/y* neurons compared with WT. ***D***, Maximum *I*_K-slow_ at the soma of *fmr1/y* neurons is less compared with WT neurons. ***E***, At the soma, maximum *I*_K-sustained_ is greater in *fmr1-/y* PT neurons compared with WT. ***F***, Summary of somatic differences in *I*_K_ between WT and *fmr1-/y* neurons. ***G***, There were no differences in dendritic K^+^ currents in *fmr1-/y* compared with WT neurons.

The maximum *I*_K-total_ was greater at the soma compared with the dendrite for WT and *fmr1-/y* neurons (*p* < 0.001^°^, ANOVA). However, the maximum somatic *I*_K-total_ in *fmr1-/y* neurons was larger than that in WT neurons (*p* = 0.01; Fig. 6*B* ^p^). This difference in somatic *I*_K-total_ is due, in part, to differences in both *I*_KA-fast_ and *I*_K-sustained_. We observed larger *I*_KA-fast_ and *I*_K-sustained_ at the soma (*p* = 0.05; fast, *p* = 0.05^q^) but not dendrite (sustained, *p* = 0.74; fast, *p* = 0.52^r^), of *fmr1-/y* compared with WT neurons (Fig. 6*C*,*E*). Consequently, *I*_KA-fast_ (*p* < 0.01) and *I*_K-sustained_ (*p* < 0.05) were larger at the soma compared with the dendrite in *fmr1-/y*, but not WT neurons (Fig. 6*C*,*E* ^s^). Interestingly, we also observed genotypic differences for the *I*_K-slow_. *I*_K-slow_ was smaller at the dendrite compared with the soma in both genotypes (*p* < 0.05^t^). In contrast to the other currents however, *I*_K-slow_ was smaller at the soma of *fmr1-/y* compared with WT neurons (*p* < 0.05; Fig. 6*D* ^u^). In summary, at the soma of *fmr1-/y* PT neurons, there is a relative increase in the contribution of *I*_KA-fast_ (57 ± 3% vs 44 ± 7%) and decrease in the contribution of *I*_K-slow_ (50 ± 6% vs 36 ± 3%) to the somatic *I*_K-total_ compared with WT (Fig. 6*F*). In contrast, we observed no differences between genotypes in relative contribution of the three K^+^ current components in the dendrites (Fig. 6*G*).

Relatively little is known about differences in K^+^ channel expression at the soma of different projection neurons in L5. Therefore, we also made outside-out patch recordings from IT neurons in mPFC to test whether the change in magnitude and relative contribution of K^+^ currents observed at the soma of PT neurons is common to all pyramidal neurons within a brain region. Similar to PT neurons, the current elicited by step depolarization to 50 mV from a holding potential of −90 mV contained both transient and sustained components. In contrast to PT neurons however, we did not observe differences in any *I*_K_ component between *fmr1-/y* and WT IT neurons at the soma or dendrite (Fig. 7; *p* > 0.05, all comparisons^v^). Thus, in the *fmr1-/y* mouse, the maximum amplitude of K^+^ currents at the soma is affected in one type of projection neuron (PT), but not a neighboring type (IT). These data suggest that channel phenotypes in the *fmr1-/y* mouse can vary not only between brain regions (ie, hippocampus, somatosensory cortex and mPFC), but also within a brain region in different classes of principal neurons (PT vs IT).

**Figure:**
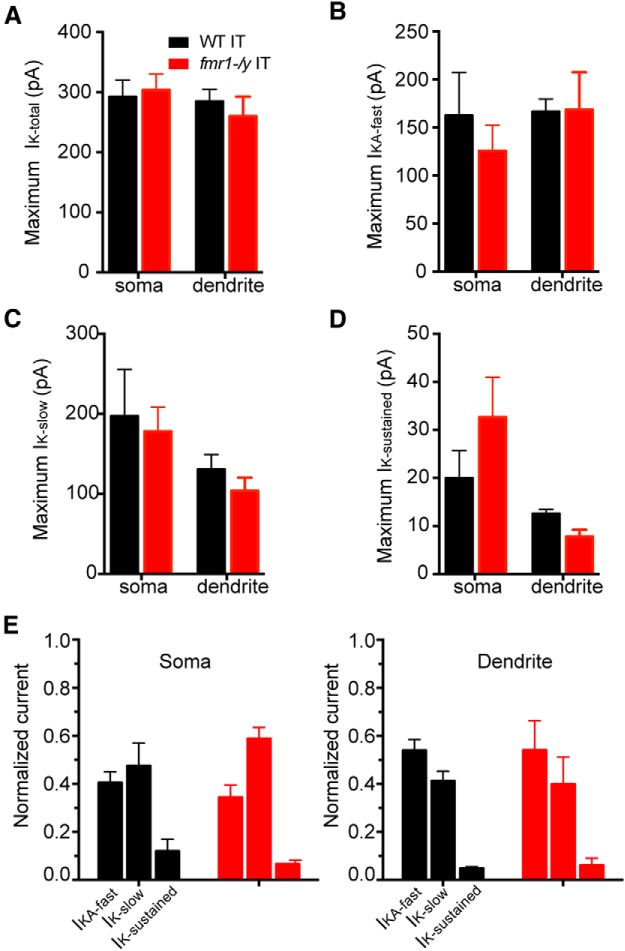
Figure 7**. *I*_K_ is not different in IT neurons in *fmr1-/y* versus WT mice.** At the soma and dendrite there were no differences in the maximum amplitude of (***A***) *I*_K-total_, (***B***) *I*_KA-fast_, (***C***) *I*_K-slow_, or (***D*)** *I*_K-sustained_ in *fmr1-/y* versus WT IT neurons. ***E***, Summary of somatic and dendritic *I*_K_ in WT (*n* = 18 somas from 12 mice and 5 dendrites from 5 mice; 180–330 µm, 250 ± 20.1 µm from soma) and *fmr1-/y* IT (*n* = 11 somas from 8 mice and 5 dendrites from 5 mice; 170–270 µm, 215 ± 27.7 µm from soma) neurons.

A-type K^+^ channels can be composed of either Kv4 or Kv1 subunits (Coetzee et al., 1999). To identify the putative channels underlying *I*_KA-fast_ and *I*_K-slow_, we used two complementary approaches. First, we exploited differences in the kinetic properties of these channels. Kv1 containing channels tend to inactivate and recover from inactivation an order of a magnitude slower than Kv4 containing channels (Castellino et al., 1995; Jerng et al., 2004). The time constant of inactivation (τ_inactivation_) of the *I*_KA-fast_ was not different between WT and *fmr1-/y* for either PT (WT τ_inactivation_ = 29 ms; *fmr1-/y* τ_inactivation_ = 25 ms) or IT neurons (WT τ_inactivation_ = 28 ms; *fmr1-/y* τ_inactivation_ = 23 ms; *p* = 0.88, ANOVA^w^). These values were consistent with Kv4 channels. The τ_inactivation_ of the *I*_K-slow_ was an order of magnitude larger than the τ_inactivation_ of the *I*_KA-fast_ in both WT and *fmr1-/y* for PT and IT neurons, consistent with Kv1 channels. A closer comparison revealed that the τ_inactivation_ of the *I*_K-slow_ in *fmr1-/y* PT neurons was significantly smaller compared with WT PT neurons (WT τ_inactivation_ = 646 ± 95 ms; *fmr1-/y* τ_inactivation_ = 244 ± 44 ms; *p* = 0.01^x^). By contrast, there was no statistically significant difference in τ_inactivation_ for *I*_K-slow_ in IT neurons (WT τ_inactivation_ = 630 ± 100 ms; *fmr1-/y* τ_inactivation_ = 425 ± 69 ms; *p* > 0.05, Fig. 8*A* ^y^). Next, we used a two-pulse protocol to measure the recovery from inactivation. We found that the time course of recovery from inactivation was well fit by two exponentials consistent with two types of transient K^+^ channels. Both the fast (*p* = 0.2, ANOVA^z^) and slow (*p* = 0.5, ANOVA^a′^) time constant of recovery from inactivation were similar in WT and *fmr1-/y* for L5 PT and IT neurons and were consistent with Kv4-containing and Kv1-containing channels, respectively (Fig. 8*B*–*D*).

**Figure:**
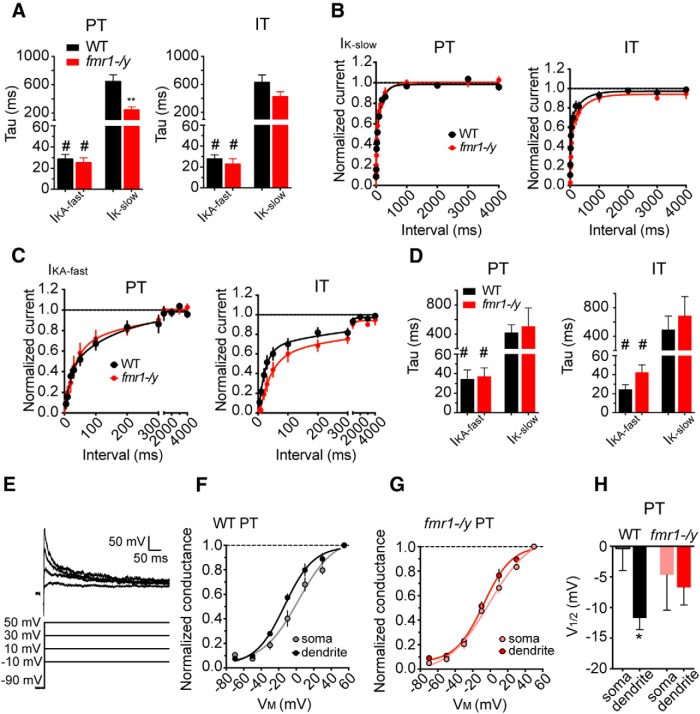
Figure 8**. Kinetic properties of K^+^ channels in L5 neurons of PFC. *A***, The time constant of inactivation of *I*_K-slow_ is significantly longer than *I*_KA-fast_ in both PT (left) and IT (right) neurons. #*p* < 0.05. Additionally, in PT neurons, the time constant of inactivation of *I*_KA-slow_ was shorter in *fmr1-/y* neurons compared with WT. *p < 0.01. ***B***, Amount of I_K-slow_ recovered from inactivation, normalized to maximum current, in PT (left) and (right) IT neurons. ***C***, Amount of *I*_KA-fast_ recovered from inactivation, normalized to maximum current, in PT (left) and IT neurons (right). ***D***, Time constant for recovery from inactivation data plotted in ***B*** and ***C*** for PT (left) and IT (right) neurons. #*p* < 0.05 *post hoc* comparison between current types in same genotype. ***E***, Protocol for measuring voltage dependence of *I*_KA_. ***F***, In WT neurons, the steady-state activation curve is shifted to hyperpolarized potentials in the dendrite relative to the soma. ***G***, In contrast, there is no difference in the activation curves between the soma and dendrite in *fmr1-/y* neurons. ***H***, the *V*_1/2_ of activation is hyperpolarized in the dendrite compared with the soma in WT neurons. In contrast, there is no difference in the *V*_1/2_ of activation between the soma and dendrite in *fmr1-/y* neurons.

As an additional method of identifying the channels underlying these currents, we exploited differences in the sensitivity of Kv1 and Kv4 containing channels to K^+^ channel blockers. Specifically, we measured the sensitivity of macroscopic K^+^ currents to 50 μm 4-aminopyridine (4-AP) and 150 μm Ba^2+^, which preferentially block K_V_1-containing and K_V_4-containing channels, respectively (Castle et al., 1994; Russell et al., 1994; Stephens et al., 1994; Coetzee et al., 1999; Gasparini et al., 2007). Additionally, we measured the sensitivity of these currents to alpha-dendrotoxin (α-DTX), which preferentially blocks Kv1.1-, 1.2-, and 1.6-containing channels (Harvey, 2001). 4-AP (50 μm) and α-DTX (200 nm) decreased *I*_KA- slow_ without significantly affecting *I*_KA-fast_ (*p* < 0.01, *n* = 8 patches from 4 mice and *p* < 0.001, *n* = 5 patches from 4 mice, respectively; two-way RM ANOVA; Fig. 9*A*,*B* ^b′^). In contrast, 150 μm Ba^2+^ decreased *I*_KA –fast_ without significantly affecting *I*_KA-slow_ (*p* < 0.005, *n* = 5 patches from 3 mice; two-way RM ANOVA; Fig. 9*C* ^c′^). Furthermore, the amplitude of the DTX- and 4-AP-sensitive current was similar to the amplitude of *I*_KA-slow_, whereas the amplitude of the Ba^2+^-sensitive current was similar to the amplitude of *I*_KA-fast_ (Fig. 9*D*). Together, these data suggest that Kv4 containing channels contribute to *I*_KA-fast_ and Kv1 containing channels contribute to *I*_K-slow_. Thus, our data suggest that in the *fmr1-/y* mouse, there are alterations to the functional expression of two K^+^ channels in PT L5 neurons of mPFC; there is an increase in the expression of Kv4-containing channels and a decrease in Kv1-containing channels.

**Figure:**
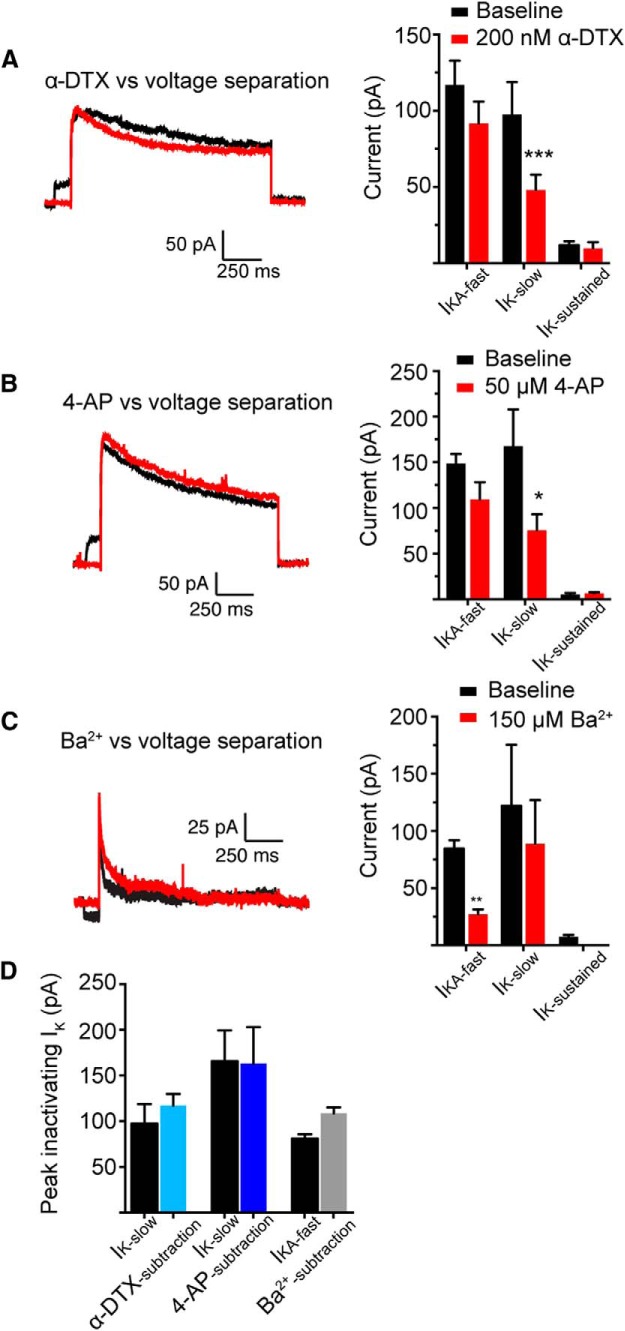
Figure 9**. Sensitivity of K^+^ currents to K^+^ channel blockers. *A***, Sample sweeps recorded from the same patch comparing the α-DTX-sensitive current to *I*_K-slow_. α-DTX (200 nm) affected *I*_K-slow_, but not other K^+^ currents. ***B***, Sample sweeps comparing the 4-AP-sensitive current to *I*_K-slow_. 4-AP (50 μm) affected *I*_K-slow_, but not other K^+^ currents. ***C***, Sample sweeps comparing the Ba^2+^-sensitive current to *I*_KA-fast_. Ba^2+^ (150 μm) affected *I*_K-slow_, but not other K^+^ currents. ***D***, Summary of currents obtained via voltage separation versus K^+^ channel blockers. The amplitude of 4-AP and α-DTX-sensitive current is similar to the amplitude of *I*_K-slow_ obtained via voltage separation, whereas the amplitude of the Ba^2+^-sensitive current is similar to *I*_KA-fast_.

In addition to a decrease in channel expression, there is a hyperpolarizing shift in the voltage dependence of activation of Kv4 channels in CA1 pyramidal neuron dendrites of *fmr1-/y* mice (Routh et al., 2013). Thus, although there is a decrease in total maximum current, there are relatively more channels open at hyperpolarized potentials in *fmr1-/y* mice compared with WT mice. To test for similar alterations to channel function in L5 neurons of mPFC, we measured the voltage dependence of activation of fast A-type K^+^ channels in somatic and dendritic patches from WT and *fmr1-/y* mice (Fig. 8*E*). Consistent with published reports in granular cortex (Bekkers, 2000), there was a hyperpolarizing shift in the activation curve of *I*_KA-fast_ at the dendrite compared with the soma in WT L5 PT neurons (*p* = 0.03; Fig. 8*F*,*H* ^d′^). In contrast, there was no such shift in *fmr1-/y* neurons (*p* = 0.76; Fig. 8*G*,*H* ^e′^). Furthermore, there was no difference in slope factor between WT and *fmr1-/y* neurons at either recording location (*p* > 0.3^f′^, ANOVA). Thus, the hyperpolarizing shift in the activation of *I*_KA-fast_ normally observed from soma to dendrite in WT neurons is absent in *fmr1-/y* neurons. These data suggest that although there is no difference in maximum *I*_KA-fast_ in the dendrites of *fmr1-/y* versus WT PT neurons, there may subtle differences in voltage dependence of activation.

### Altered action potential generation in *fmr1-/y* L5 PT neurons of mPFC

K^+^ channels modulate several aspects of neural function, including the dynamics of action potential threshold. Changes to threshold, also known as threshold accommodation, can be mediated through the recruitment of K^+^ channels, in addition to Na^+^ channel inactivation (Hodgkin and Huxley, 1952). Kv4 and Kv1 subunits have both been implicated in regulating neural excitability by setting the threshold for action potential generation (Bekkers and Delaney, 2001; Kim et al., 2005; Higgs and Spain, 2011; Carrasquillo et al., 2012). Because we observed somatic differences consistent with alterations to these two K^+^ channels, we predicted that threshold dynamics would be altered in L5 PT neurons in mPFC.

To test this hypothesis, we injected current of varied duration at the soma of L5 PT neurons in both genotypes (WT: *n* = 20 cells from 11 mice; *fmr1-/y*: *n* = 23 cells from 9 mice). The current amplitude for a given duration was adjusted to be just-threshold for the generation of an action potential (Fig. 10*A*). Action potential threshold varied as a function of current duration in both genotypes (*p* < 0.001, ANOVA^g′^). However, for durations greater than 6 ms, spike threshold was significantly more hyperpolarized in *fmr1-/y* PT neurons compared with WT neurons (*p* < 0.01; *post hoc* comparisons^h′^; Fig. 10*A*–*C*). Furthermore, the current required to generate an action potential was significantly lower in *fmr1-/y* compared with WT neurons at all current durations (*p* < 0.001, ANOVA^i′^). Differences in Na^+^ channel availability can produce differences in action potential threshold (Hodgkin and Huxley, 1952). However, there was no significant difference in the maximum rate of rise of action potentials in WT versus *fmr1-/y* neurons, a spike parameter sensitive to Na^+^ channel availability (*p* = 0.30; ANOVA^j′^; Colbert et al., 1997). There was also no difference in action potential amplitude between genotypes (*p* = 0.59; ANOVA). Finally, there were no differences in action potential threshold between WT and *fmr1-/y* IT neurons (*p* = 0.31; ANOVA; Fig. 10*D* ^k′^). Together with our outside-out patch recordings, these data suggest that differences in K^+^ channel expression in PT, but not IT neurons, contribute to alterations in spike threshold dynamics in the *fmr1-/y* mouse mPFC, resulting in an increase in excitability.

**Figure:**
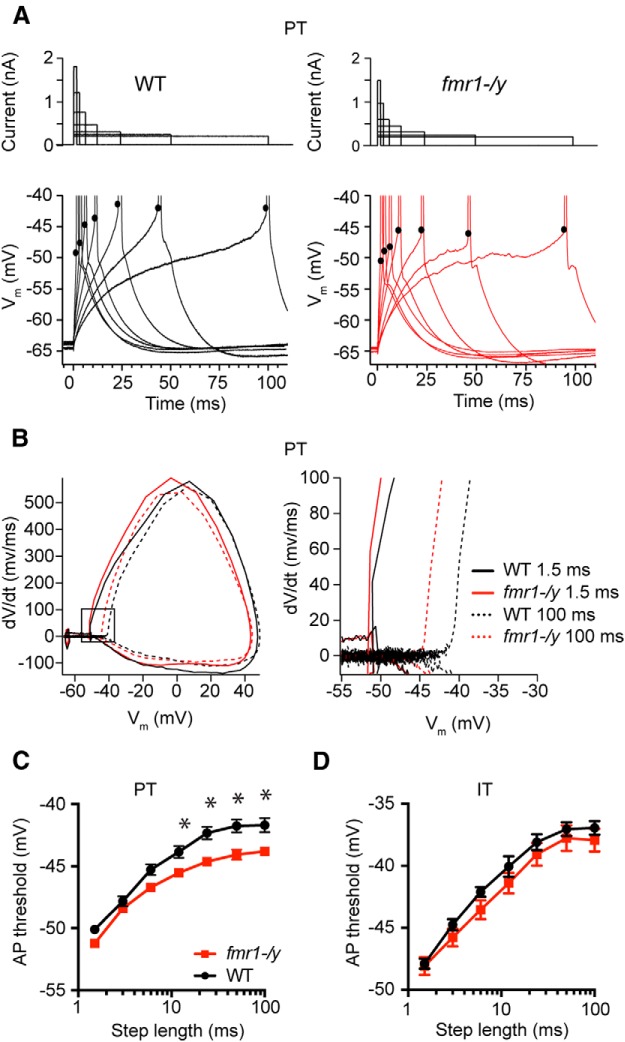
Figure 10**. *Fmr1-/y* PT neurons are more excitable compared with WT neurons. *A***, The threshold for action potential generation in WT and *fmr1-/y* neurons was measured in response to just threshold current injections of increasing duration. Sample action potentials for each genotype are shown below. ***B***, Phase plane plot of action potentials in *fmr1-/y* and WT PT neurons in response to 1.5 and 100 ms current injection. ***C***, *fmr1-/y* neurons displayed a lower threshold for action potential generation for all current injections longer than 6 ms. ***D***, There were no differences in threshold between *fmr1-/y* and WT IT neurons. *p < 0.01 comparison between genotypes.

As an additional test that differences in K^+^ channel function contribute to differences in excitability, we measured the sensitivity of threshold to 50 μm 4-AP and 150 μm Ba^2+^, and 200 nm α-DTX. Bath application of 50 μm 4-AP produced a decrease in threshold for all but the shortest current durations in both genotypes (WT and *fmr1-/y*: *n* = 6 cells from 4 mice; *p* < 0.01, ANOVA^l′^). This effect, however, was larger in WT compared with *fmr1-/y* neurons, consistent with a lower contribution of K_V_1-containing channels and in agreement with our current measurements (*p* < 0.01; Fig. 11*A* ^m′^). Similarly, 200 nm α-DTX produced a larger affect on threshold in WT compared with *fmr1-/y* neurons (WT and *fmr1-/y: n* = 5 cells from 4 mice; *p* = 0.02; ANOVA; Fig. 11*B* ^n′^). Ba^2+^ (150 μm) produced a small but significant decrease in threshold for all but the shortest current durations in both genotypes (WT and *fmr1-/y*: n = 8 from 4 and 5 mice, respectively; *p* < 0.01; ANOVA; Fig. 11*C* ^o′^). Interestingly, in WT mice the effect of 4-AP and α-DTX was larger than that of Ba^2+^ (*p* < 0.001, ANOVA^p′^), whereas in *fmr1-/y* mice there was no difference in the effect of these K^+^ channel blockers (*p* = 0.55, ANOVA; Fig. 11*D* ^q′^). These data suggest that in *fmr1-/y* PT neurons, a decrease in Kv1-mediated K^+^ current contributes to an increase in excitability.

**Figure:**
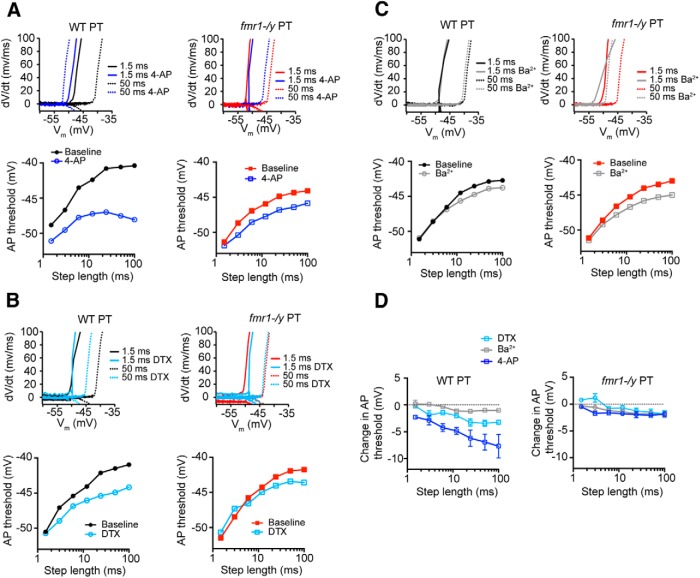
Figure 11**. *Fmr1-/y* neurons are less sensitive to** Kv**1 channel blockers compared with WT neurons. *A***, Phase plane plots for action potentials generated in response to 1.5 and 50 ms current injections before and after 50 μm 4-AP application. 4-AP decreased threshold in both genotypes, but more so in WT neurons. ***B***, Phase plane plots for action potentials generated before and after 200 nm α-DTX application. α-DTX decreased threshold in both genotypes, but more so in WT neurons. ***C***, Phase plane plots for action potentials generated before and after 150 μm Ba^2+^ application. Ba^2+^ decreased action potential threshold in both genotypes. ***D*,** 4-AP and α-DTX application had a larger effect on action potential threshold compared with Ba^2+^ in WT neurons. In contrast, 4-AP and α-DTX had the same effect on action potential threshold compared with Ba^2+^ in *fmr1-/y* neurons. For current steps longer than 6 ms, there was significant difference in the effect of 4-AP and/or α-DTX on threshold between WT and *fmr1-/y* mice.

## Discussion

We have provided evidence for neuron-type-specific channelopathies within a single brain region in FXS. We observed several differences in the functional expression of ion channels in L5 neurons of mPFC in the *fmr1-/y* mouse (Fig. 12). At the soma and dendrite, we observed differences in the subthreshold properties of *fmr1-/y* PT neurons consistent with a decrease in *I*_h_ compared with WT neurons. These differences were largely eliminated by the application of the h-channel blocker ZD7288, suggesting that they were mediated, in part, by differences in h-channels. Consistent with this, we observed less *I*_h_ at the distal dendrite of *fmr1-/y* PT neurons, suggesting that there is a reduction in functional h-channels. Additionally, we found alterations to several K^+^ channels at the soma of PT neurons. Compared with WT neurons, *fmr1-/y* neurons possessed greater maximum fast-inactivating A-type K^+^ current, but less slow-inactivating K^+^ current. The kinetics and pharmacologic sensitivity of these currents was consistent with Kv4 and Kv1 containing channels, respectively. In contrast to PT neurons, there were no genotypic differences in the maximum amplitude of these K^+^ currents at the soma of IT neurons. As a functional consequence of altered somatic K^+^ channel function in PT neurons, we observed an increase in excitability.

**Figure:**
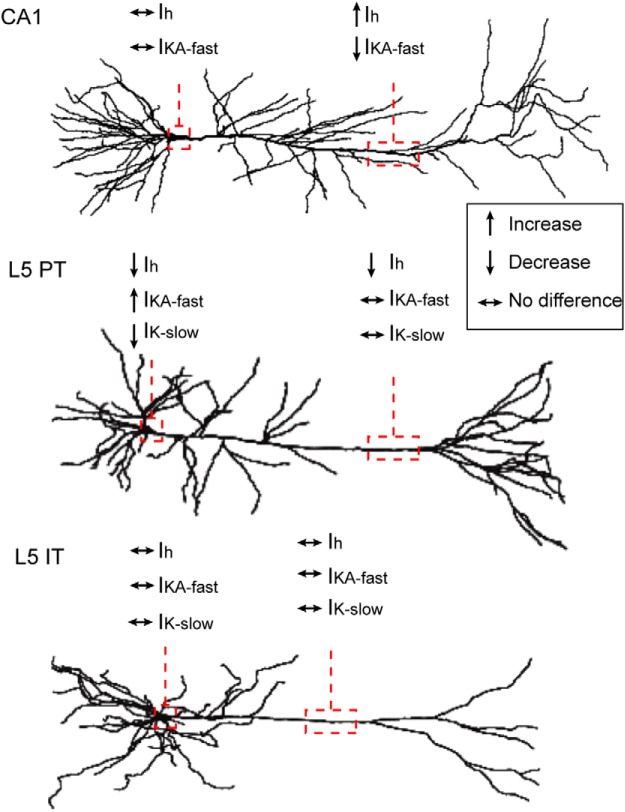
Figure 12**. Summary of neuron type-specific channel phenotypes observed in the *fmr1-/y* mouse.** Dendritic and somatic *I*_h_, *I*_KA_-fast, and *I*_K_-slow levels in the *fmr1-/y* mouse relative to WT in CA1 pyramidal neurons (Brager et al., 2012; Routh et al., 2013), L5 PT neurons of PFC and IT neurons of PFC. There is also a decrease in *I*_h_ in the dendrites of L5 neurons of somatosensory cortex (data not shown; Zhang et al, 2014). Upward arrow indicates increase relative to WT. Downward arrow indicates decrease relative to WT. Horizontal arrow indicates no difference relative to WT.

These findings expand on previous reports of channelopathies in various brain regions by demonstrating neuron specific phenotypes within a brain region (Brager and Johnston, 2014). In L5B neurons of somatosensory cortex a decrease in h-channel expression, together with altered BK channel function, contributes to a lowered threshold for dendritic Ca^2+^ electrogenesis (Zhang et al., 2014). In contrast to somatosensory cortex, there is an increase in h-channel expression in CA1 pyramidal neurons in the *fmr1-/y* mouse (Brager et al., 2012). Additionally, in CA1 pyramidal neurons, there is a decrease in Kv4-mediated A-type K^+^ current (Routh et al., 2013). Our data, together with these previously published findings, suggest that in FXS the function of a given ion channel can be affected in opposite ways depending upon brain region. More importantly, the absence of differences in K^+^ channel function in IT neurons suggests that neuron-specific channel phenotypes can occur even within a brain region, between different classes of principal neurons (Fig. 12). Alterations to other channel types reported in various brain regions may similarly display neuron-type specificity.

### Identity of K^+^ channels altered in mPFC

Kv1 and Kv4 containing channels contribute to transient K^+^ currents (Coetzee et al., 1999). Although the details depend upon which auxiliary subunits are present, Kv4 channels inactivate and recover from inactivation an order of a magnitude faster than Kv1 channels (Castellino et al., 1995; An et al., 2000; Jerng et al., 2004; Kim et al., 2008). Thus, the *I*_KA-fast_ we observed is consistent with Kv4 containing channels whereas the *I*_K-slow_ is consistent with Kv1 channels. Several subunits within the Kv1 and Kv4 families can contribute to transient K^+^ currents (Coetzee et al., 1999). There is evidence that Kv4.2, 4.3, and 1.4 contribute to transient K^+^ currents at the soma of cortical pyramidal neurons (Carrasquillo et al., 2012). In addition, Kv1.2 containing channels contribute to a slowly inactivating K^+^ current at the soma (Bekkers and Delaney, 2001). All of these subunits have been reported to contribute to action potential threshold and thus alterations to any of these subunits could contribute to differences in *I*_K_ and excitability we observe (Bekkers and Delaney, 2001; Kim et al., 2005; Kole et al., 2007; Higgs and Spain, 2011). Regardless of the subunit, the differences in maximum current we observed are consistent with a decrease in the number of Kv1 channels and an increase in Kv4 channels in PT neurons of mPFC in the *fmr1-/y* mouse.

### Potential mechanisms

Brain region-specific channel phenotypes in FXS could be caused by a primary deficiency in an FMRP-mediated process within the cell. FMRP is an mRNA binding protein and has been shown to negatively regulate protein synthesis through ribosomal stalling (Li et al., 2001; Zalfa et al., 2003; Darnell et al., 2011). Consequently, the absence of FMRP could lead to elevated expression of ion channel proteins and/or their regulatory subunits. More recent evidence suggests that FMRP can also promote the translation of certain mRNAs (Bechara et al., 2009; Fähling et al., 2009; Gross et al., 2011). Thus, the absence of FMRP might then reduce the expression of ion channel proteins. In addition to its role in translation, FMRP participates in the activity-dependent transport of mRNA granules from the soma to axonal and dendritic locations (Antar et al., 2004; Kanai et al., 2004; Dictenberg et al., 2008). The absence of FMRP could therefore result in the trapping of ion channel mRNAs at the soma or the mislocalization of mRNAs and the encoded proteins. Both h-channel and A-type K^+^ channel mRNAs are known targets of FMRP (Gross et al., 2011; Lee et al., 2011; Darnell et al., 2011). Therefore, the channel phenotypes observed in FXS could be because of either of these mRNA regulatory processes. Lastly, FMRP can directly bind to target proteins, including ion channel subunits. Consequently, the absence of FMRP could alter the biophysical properties or surface expression of ion channels by promoting inappropriate subunit assemblies (Brown et al., 2010; Deng et al., 2013). It is noteworthy that in order to sufficiently explain the divergent channel phenotype we observe, these cellular processes would have to promote or suppress the function of a given ion channel in a neuron-specific manner.

Alternatively, the differences in channel function we observe could be the result of secondary changes in response to altered processes extrinsic to the cell. Both *I*_h_ and *I*_KA_ undergo synaptic-driven changes (Frick et al., 2004; Fan et al., 2005; Brager and Johnston, 2007; Losonczy et al., 2008). Thus, a potential cell extrinsic mechanism could involve changes in synaptic input to mPFC neurons that result in changes in ion channel function opposite to those that occur in CA1. Indeed there is evidence that synaptic input to neocortical and hippocampal pyramidal neurons is altered in the *fmr1-/y* mouse (Bureau et al., 2008; Gibson et al., 2008; Hays et al., 2011; Krueger et al., 2011; Testa-Silva et al., 2012; Patel et al., 2013; Tyzio et al., 2014). In addition, the function of both *I*_h_ and *I*_KA_ are affected by neuromodulation (Hoffman and Johnston, 1999; Carr et al., 2007; Dembrow et al., 2010). Thus, the divergent channel phenotypes observed in CA1 and neocortex could be caused by differences in neuromodulation. Determining whether neuron-type-specific alterations to ion channel function are caused by a cell autonomous or cell extrinsic mechanism (eg, differences in network activity) is a difficult challenge, yet fundamental to our understanding of the neurobiology of FXS.

### Functional implications

The combined effects of the channel phenotypes we observe could have a substantial impact on mPFC function. PT neurons receive synaptic input in L1 from various sources and the presence of *I*_h_ greatly influences the transfer of these inputs to the soma (Dembrow et al., 2015). Specifically, *I*_h_ contributes to the filtering properties of the membrane such that inputs arriving in the theta frequency range are selectively transferred to the soma (Ulrich, 2002; Cook et al., 2007). Additionally, *I*_h_ compensates for distance dependent temporal delays imposed by the dendrite, ensuring that distal inputs arrive at the soma with a similar latency to proximal ones (Vaidya and Johnston, 2013). This has the effect of narrowing the temporal window under which synaptic input arriving at different dendritic locations can summate at the soma to drive an action potential (Dembrow et al., 2015). Thus, the decrease in *I*_h_ we observe in the *fmr1-/y* mouse could: (1) affect the frequency preference of PT neurons to synaptic input, and (2) increase integration at the soma of asynchronous dendritic synaptic inputs. Consequently, *fmr1-/y* PT neurons may function as temporal integrators of synaptic input, in contrast with WT PT neurons, which function as coincidence detectors (Dembrow et al., 2015).

These changes in *I*_h_ could work synergistically with K^+^ channel alterations to make *fmr1-/y* PT neurons generally more responsive to synaptic input. K^+^ channels affect the threshold for spike generation in part by influencing the voltage at the axon initiation site (Bekkers and Delaney, 2001; Kole et al., 2007). Like h-channels, K^+^ channels contribute to the band pass filtering properties of the membrane. Thus, the changes in K^+^ channels we observe could also decrease the preference of PT neurons for high-frequency input as has been observed upon blockade of Kv1 channels in L2/3 neurons (Higgs and Spain, 2011). Together with changes to synaptic input, these changes may contribute to altered up-states observed in cortical pyramidal neurons in the *fmr1-/y* mouse (Hays et al., 2011; Gonçalves et al., 2013).

FXS patients display behavioral deficits associated with PFC dysfunction, including hyperactivity, attention deficits, impulsivity, and behavioral inflexibility. The channel phenotypes we describe here may thus contribute to these behavioral phenotypes. Finally, our findings of cell-type-specific phenotypes highlights the challenges associated with developing effective treatment strategies in FXS. A critical implication of our findings is that existing therapies that target a given channel phenotype, although effective in treating one feature of FXS could simultaneously exacerbate others. Understanding the mechanisms of these neuron/brain region-specific effects in FXS may prove critical in developing treatment strategies.

## Synthesis

The decision was a result of the Reviewing Editor Zoltan Nusser and the peer reviewers coming together and discussing their recommendations until a consensus was reached. A fact-based synthesis statement explaining their decision and outlining what is needed to prepare a revision is listed below.

The present MS examined changes in Ih and IA in two distinct layer 5 pyramidal cell populations (PT and IT) in fmr1-/y mice. These mice have been extensively used and are considered as one of the best animal models of Fragile-X syndrome. In general, the electrophysiological recordings have been carefully designed and are of high quality. However, there are two major issues regarding the MS: one is the novelty and the second is the lack of mechanistic understanding of the observed changes. Both reviewer found that the MS is rather a logical continuation of previous works and it can be considered an incremental step. Brain region-specific changes in the excitability of pyramidal cells in fmr1-/y mice have been demonstrated, therefore the fact the MPFC pyramidal cells behave differently compared to those in the somatosensory cortex or hippocampus is not surprising. The finding that PT and IT neurons in the PFC also regulated differentially has more novelty. Here it is important to note that that data from anterior cingulate and prelimbic areas of the PFC should not be pooled together. The authors should re-analyze their data and separate cells from these two subregions. The second major issue is much more fundamental and it is extremely hard to provide any evidence that the observed changes are the consequence of the lack of Fmr1 protein in the examined cells or it is due to changes i.e. in network excitability. This should be openly discussed in the MS. Detailed comments from the reviewers are listed below:

MAJOR:

1 Data obtained from neurons recorded in both anterior cingulate and prelimbic areas of the PFC were pooled. If the main message of the article is that differences of K+ channels within the same brain region are biologically relevant, it is not appropriate to pool data from two functionally distinct brain regions.

2 The patch-clamp data are presented per individual cell. The individual variable is the animal (and the PFC sub region see previous comment) not the individual cell.

3 Figure 1A shows colocalized (yellow) IT and PT neurons. Why is it concluded that there is no overlapping? Here also, the number of animal tested is too small (l. 180: n=2)

MINOR:

- Please correct typos and inconsistencies in the reference list (e.g. l. 734, 720, 651 & 689).

- It is not clear why the recent papers by the Sohal group showing cell subtypes based on their connectivity and expression of D1 or D2 dopamine receptors are not cited and discussed.

MAJOR:

1) Throughout the manuscript the author(s) contrast their findings with previous work in the CA1 pyramidal cells. (Line 11: "this is the opposite pattern", line 55 "in contrast to hippocampus", line 60 "in contrast to hippocampus", etc.). The authors even include in Figure 12 a graphical summary by showing CA1 compared with the mPFC changes. This implies a novelty effect which is misleading. The comparison is weak for the reason that the authors could also have selected the results from Zhang et al. Nat Neurosci., 2014 which reported a HCN channel reduction in dendrites from somatosensory neocortical pyramidal neurons. The manuscript would benefit from a more focused writing and discussion about the distinct responses in the PT and IT cell types.

2) Figure 5A. The pharmacological isolation of the HCN-current seems inadequate. At -30 mV during deactivation there is a fast outward current in the control tail current but not in fmr1. These distinct tail currents cannot be explained by a distinct transient Kv channel expression in Fmr1 (Figure 6). Stepping to -60 mV would have been better. Were currents sensitive to ZD7288? Please clarify.

MINOR:

- Line 114. How can the pipette capacitance and bridge balance be compensated before starting a whole-cell recording before assessment of the residual transients? Assuming this is a typo, change "before" into "during".

- Line 147. Potentials more hyperpolarized than -10 mV? Please clarify or correct. The procedure of fitting is not clear.

- Figure 1A. Colours should be labelled in the figure and a schematic of the region would be helpful.

- Figure 1C. Use closed opaque filling of diamonds. The IT symbols are not much different from the PT symbols.

- Figure 3A. delta X is not defined.

- At which distances were the data from Fig. 5A obtained?

- Line 262. Figure 5D. The correlation between resonant frequency and H-current is flawed. It was already shown that the average frequency and Ih densities are different between groups (Figures 2 and 5). The correlation would gain in strength, however, if Ih amplitudes would be shown as a scatter plot of the individual dendritic recordings.

- Figure 10B. The phase-plane examples show a reduced AP amplitude. Is this representative and/or are the averages different? Please report the average values in the results (threshold-to-peak amplitudes).

- Figures 10C, D and Figures 11. The X-axes are not scaled.

- Figure 11D. Please indicate for which time points there were differences between control and fmr1.

- Citation to Brager et al., 2012 is incomplete.

## Author Response

We thank the reviewers for their thoughtful comments. Addressing these comments has resulted in a stronger manuscript. Please find below a point-by-point reply to the comments.

Synthesis Statement for Author (Required):

The present MS examined changes in Ih and IA in two distinct layer 5 pyramidal cell populations (PT and IT) in fmr1-/y mice. These mice have been extensively used and are considered as one of the best animal models of Fragile-X syndrome. In general, the electrophysiological recordings have been carefully designed and are of high quality. However, there are two major issues regarding the MS: one is the novelty and the second is the lack of mechanistic understanding of the observed changes. Both reviewer found that the MS is rather a logical continuation of previous works and it can be considered an incremental step. Brain region-specific changes in the excitability of pyramidal cells in fmr1-/y mice have been demonstrated, therefore the fact the MPFC pyramidal cells behave differently compared to those in the somatosensory cortex or hippocampus is not surprising.

- We would like to make two points here about the novelty of our work. First, although Ih has been reported to be upregulated or downregulated in fmr1-/y mice depending on brain region, IA has not. Indeed, how IA is affected in fmr1-/y mice is controversial with some arguing that it is increased (e.g. Lee et al) and others reporting that it is decreased (Gross et al, Routh et al). Our data suggest that the effect of the absence of FMRP on IA, like Ih, depends on the brain region and even the principal neuron type within a brain region. Furthermore, we report for the first time cell-type specific differences in a Kv1 like channel. We argue that these are novel findings and hardly incremental. Second, our findings might help convince the field that brain region specific differences in the function of a single type of ion channel are a real phenomenon and not caused by a trivial difference (e.g., differences in preparations, ages of mice, background strains, etc.). When separate labs observe opposite effects it is often a recipe for disagreement. The fact that we, a single lab, have observed in the fmr1-/y mouse Ih increase in CA1 but decrease in mPFC should help quell potential disagreement.

The finding that PT and IT neurons in the PFC also regulated differentially has more novelty. Here it is important to note that that data from anterior cingulate and prelimbic areas of the PFC should not be pooled together. The authors should re-analyze their data and separate cells from these two subregions. The second major issue is much more fundamental and it is extremely hard to provide any evidence that the observed changes are the consequence of the lack of Fmr1 protein in the examined cells or it is due to changes i.e. in network excitability. This should be openly discussed in the MS.

- If the reviewers mean to say that we should openly discuss potential mechanisms of neuron-type specific alterations, we have already done so in the discussion. We gave a detailed description of potential cell autonomous and network mechanisms in two sequential paragraphs. We have also now acknowledged that discriminating between these two general mechanisms is a difficult yet worthy pursuit.

Detailed comments from the reviewers are listed below:

Reviewer 1

1/ Data obtained from neurons recorded in both anterior cingulate and prelimbic areas of the PFC were pooled. If the main message of the article is that differences of K+ channels within the same brain region are biologically relevant, it is not appropriate to pool data from two functionally distinct brain regions.

- The original intent of this sentence was to indicate that data were obtained from the dorsal regions of mPFC ~1-2 mm anterior to bregma. Most of these recordings were at the border of anterior cingulate and prelimbic cortex where it is difficult to discriminate between these two brain regions (i.e. there are no clear anatomical or cytoskeletal distinctions). We have changed this sentence in the methods to more accurately reflect where recordings were obtained, but unfortunately we cannot separate data based on prelimbic/anterior cingulate.

2/ The patch-clamp data are presented per individual cell. The individual variable is the animal (and the PFC sub region see previous comment) not the individual cell.

- We now include the number of animals used in each comparison.

3/ Figure 1A shows colocalized (yellow) IT and PT neurons. Why is it concluded that there is no overlapping?

- This experiment was performed to supplement the physiological recordings, which clearly indicate that IT and PT neurons do not overlap. Nonetheless, after looking through several 300 ¼m thick slices from each animal we concluded that there is no obvious overlap between red and green beads. The areas that the reviewer is referring to may either be auto fluorescence or reflect light from out of focus neurons. To our eye, there are no clear areas where red and green puncta overlap at a given plane of focus. We have added a sentence to clarify this in the results as well as a sentence in the methods to better describe this process.

Here also, the number of animal tested is too small (l. 180: n=2)

- This n is consistent other published results in the field (e.g. Dembrow et al 2010; Hattox and Nelson, 2007; Morishima and Kawaguchi, 2006) and thus we feel it is sufficient. Furthermore, as we noted above, these experiments were performed to supplement the physiological recordings, which clearly suggest IT and PT neurons are non-overlapping (Fig 1c).

Please correct typos and inconsistencies in the reference list (e.g. l. 734, 720, 651 & 689).

- We have corrected these and thank the reviewer for alerting us to them.

It is not clear why the recent papers by the Sohal group showing cell subtypes based on their connectivity and expression of D1 or D2 dopamine receptors are not cited and discussed.

- We apologize for the omission and now include these citations.

Reviewer 2

1) Throughout the manuscript the author(s) contrast their findings with previous work in the CA1 pyramidal cells. (Line 11: “this is the opposite pattern", line 55 “in contrast to hippocampus", line 60 “in contrast to hippocampus", etc.). The authors even include in Figure 12 a graphical summary by showing CA1 compared with the mPFC changes. This implies a novelty effect which is misleading. The comparison is weak for the reason that the authors could also have selected the results from Zhang et al. Nat Neurosci., 2014 which reported a HCN channel reduction in dendrites from somatosensory neocortical pyramidal neurons. The manuscript would benefit from a more focused writing and discussion about the distinct responses in the PT and IT cell types

- We have made changes to avoid the impression that brain region specific alterations to Ih is a novel finding. We have added the reference to the Zhang paper when discussing Ih alterations in other brain regions. However, we include only CA1 and mPFC in the graphical summary for a couple of reasons. 1- It is not clear from which neuron type Zhang et al were recording (e.g IT, PT or both) and 2- for concision, because the Zhang paper did not explore differences in the K+ currents we recorded.

2) Figure 5A. The pharmacological isolation of the HCN-current seems inadequate. At -30 mV during deactivation there is a fast outward current in the control tail current but not in fmr1. These distinct tail currents cannot be explained by a distinct transient Kv channel expression in Fmr1 (Figure 6). Stepping to -60 mV would have been better. Were currents sensitive to ZD7288? Please clarify.

- We measured only the peak of the inward current and no efforts were made to measure the tail currents. We observed this outward tail current in both genotypes, and it likely reflects the activation of Kv channels and thus would not affect our measurement of Ih.

Line 114. How can the pipette capacitance and bridge balance be compensated before starting a whole-cell recording before assessment of the residual transients? Assuming this is a typo, change “before” into “during".

- We have made this correction

Line 147. Potentials more hyperpolarized than -10 mV? Please clarify or correct. The procedure of fitting is not clear.

- We have made this clearer.

- Figure 1A. Colours should be labelled in the figure and a schematic of the region would be helpful.

- We have labeled colors and include a sample coronal section of PFC to help orient the reader.

- Figure 1C. Use closed opaque filling of diamonds. The IT symbols are not much different from the PT symbols.

- IT symbols are now filled.

- Figure 3A. delta X is not defined.

- These distances are now included in the main body of the text

- At which distances were the data from Fig. 5A obtained?

- These recordings were done at 225-400 µm, 340 ± 37.6 µm from soma for WT and 250-350 µm, 308 ± 17.7 µm from soma for KO. These values are now included in the text

- Line 262. Figure 5D. The correlation between resonant frequency and H-current is flawed. It was already shown that the average frequency and Ih densities are different between groups (Figures 2 and 5). The correlation would gain in strength, however, if Ih amplitudes would be shown as a scatter plot of the individual dendritic recordings.

- We did not mean to imply that there was a statistical correlation between these measurements. This figure was only meant to be a succinct summary showing resonance and Ih are reduced in the same fmr1-/y neurons. We have eliminated text that might be misinterpreted as suggesting statistical correlation.

- Figure 10B. The phase-plane examples show a reduced AP amplitude. Is this representative and/or are the averages different? Please report the average values in the results (threshold-to-peak amplitudes).

- The average AP amplitude is not different between genotypes. We now report this in the text.

- Figures 10C, D and Figures 11. The X-axes are not scaled.

- We have corrected this.

Figure 11D. Please indicate for which time points there were differences between control and fmr1.

- We have added a sentence in the figure legend to clarify this.

- Citation to Brager et al., 2012 is incomplete.

- We have corrected this citation.
